# Integrating Mediterranean Diet Education and Sustainability in Primary Schools: A Two-Season Community-Based Intervention in Portugal

**DOI:** 10.3390/nu18152405

**Published:** 2026-07-23

**Authors:** Rute F. Vitor, Vanda Lopes de Andrade, João Reis, Miguel Macário, Igor Dias, Maria Figueiredo, Maria Rodrigues, Laura Mendes, Inês Ferrão, Rafael Barros, Paula Ruivo

**Affiliations:** 1 School of Agriculture, Santarém Polytechnic University, Quinta do Galinheiro—S. Pedro, 2001-904 Santarém, Portugal; vanda.andrade@esa.ipsantarem.pt (V.L.d.A.); joao.reis@esa.ipsantarem.pt (J.R.); miguel.macario@esa.ipsantarem.pt (M.M.); igor.dias@esa.ipsantarem.pt (I.D.); mariadacostafigueiredo@gmail.com (M.F.); marialb.rodrigues@hotmail.com (M.R.); laurasmendes2001@hotmail.com (L.M.); inesfsf060818@gmail.com (I.F.); rafaelfbarros6@gmail.com (R.B.); 2 Life Quality Research Centre (CIEQV), Santarem Polytechnic University, Complexo Andaluz, Apartado 279, 2001-904 Santarém, Portugal; 3 Research Centre for Natural Resources, Environment and Society (CERNAS), Santarém Polytechnic University, Quinta do Galinheiro—S. Pedro, 2001-904 Santarém, Portugal; 4 Research Institute for Medicines (iMed.ULisboa), Faculty of Pharmacy, Universidade de Lisboa, 1649-003 Lisboa, Portugal

**Keywords:** school-based interventions, Mediterranean diet, food waste, sustainable healthy diets, primary school, KIDMED index

## Abstract

**Background/Objectives**: School-based programmes may play an important role in promoting healthier and more sustainable dietary habits from early childhood. This study described and evaluated a community-based intervention aimed at promoting Mediterranean diet (MD) principles, improving food literacy, and raising awareness of sustainable food practices among primary school children in the Tagus Lezíria region, Portugal. **Methods**: The intervention was implemented in 23 schools across 11 municipalities and included two seasonal rounds of participatory educational activities focused on healthy eating, local and seasonal foods, sensory exploration, balanced meals, composting, food storage, sustainable food production, and food waste prevention. A total of 872 children participated in the intervention activities, and 370 provided paired baseline and follow-up KIDMED assessments. **Results**: The mean KIDMED score decreased significantly from 8.0 ± 2.2 at baseline to 7.5 ± 2.4 after the intervention. Overall, 31.6% of participants improved their score, 18.9% showed no change, and 49.5% showed a decrease. The proportion of children who did not skip breakfast increased from 79.8% to 88.6%, and avoidance of fast-food restaurants more than once weekly increased from 85.4% to 89.6%. Smaller favourable changes were observed for pastry, sweet, and fish consumption, and olive oil use, whereas fruit, vegetable, pulse, and nut consumption did not improve. **Conclusions**: The intervention produced heterogeneous outcomes. Although overall MD adherence decreased, almost one-third of participants improved individually, with favourable changes in breakfast and selection of less healthy behaviours. Longer programmes with stronger family involvement may be required to reinforce core MD foods and achieve broader, sustained improvements.

## 1. Introduction

The Mediterranean diet (MD), a term proposed by Ancel Keys in 1960, represents the cultural dietary practices of populations surrounding the Mediterranean Sea [1]. This dietary model is not only a hallmark of nutrition but also a reflection of social behaviours and lifestyles unique to the Mediterranean region. The MD extends beyond food choices to encompass social, cultural, economic, and environmental aspects, and is deeply rooted in the traditions of these communities. Recognized as Intangible Cultural Heritage by the United Nations Educational, Scientific, and Cultural Organization (UNESCO) since 2014, the MD highlights agricultural and food consumption practices that foster a responsible interaction with the environment emphasizing biodiversity, seasonality, and food variety. The MD prioritizes fresh, local products that respect the natural cycles of production and promote sustainability [2,3]. For UNESCO, the MD is more than a healthy, sustainable diet—it embodies a set of skills, knowledge, rituals, symbols, and traditions that strengthen social bonds. The act of sharing meals and eating together is a core component of the MD, fostering social connections and reinforcing family and community ties [4,5]. This social element, coupled with the MD’s nutritional benefits, makes it a holistic lifestyle that supports both individual well-being and community cohesion.

The EAT-Lancet Commission recognized the MD in 2019 as one of the healthiest dietary patterns worldwide, emphasizing the importance of healthy and sustainable dietary habits from early childhood, when lifelong eating behaviours are established [6]. The MD has been associated with numerous health benefits, including a reduced risk of chronic non-communicable diseases (NCDs), such as diabetes, cardiovascular diseases and obesity, since it is based on the consumption of typical Mediterranean foods, such as fish, fruit and vegetables, and white meat, among others, to the detriment of ultra-processed foods rich in salt, sugar, and (trans) fats [7]. It is also known that several mental health problems can be exacerbated by inadequate diet [8] and poor eating habits can be associated with population groups suffering from depression [9,10]. The MD allows for the intake of foods rich in fatty acids; omega-3; vitamins B, C, D and E; zinc; magnesium; polyphenols; monounsaturated fats and essential amino acids, which in a synergetic way can improve mental health [11]. Several studies have demonstrated that MD improves socialization and significantly increases physical and psychological health domains, as well as quality of life in individuals with depression symptoms [10]. Conversely, low adherence to MD might play a role in attention deficit hyperactivity disorder (ADHD) [12] and personalized nutrition-based recommendations for persons with this condition are viewed as a promising approach [13].

Despite the documented benefits of the MD, the progressive westernization of dietary patterns has led populations—especially children and adolescents—to adopt dietary patterns higher in saturated fats, refined grains, and processed foods [14,15]. This shift, termed nutritional transition, is a key factor contributing to the rising prevalence of childhood overweight and obesity [16]. Portugal, as a country with Mediterranean characteristics, has shown a troubling trend away from the traditional Mediterranean Dietary Pattern (MDP), especially among younger populations. Studies indicate a decrease in the consumption of fruits and vegetables and an increase in high-energy-density foods like snacks, sugary items, fast food, and soft drinks [17], alongside a shift toward more sedentary lifestyles [18]. The health impacts of these dietary and lifestyle changes are profound and multidimensional, underscoring an urgent need for intervention—particularly in school settings where young people can be effectively reached.

School-based interventions aimed at promoting healthier eating have already demonstrated positive effects on children’s health parameters [19]. Research shows that nutrition education programmes in schools can significantly improve children’s dietary habits and contribute to healthier outcomes [20,21,22]. Additionally, when nutritional education is combined with programmes promoting physical activity, the benefits are even more pronounced: overweight and obese children participating in intervention programmes have shown marked improvements in both nutritional behaviour and Mediterranean Diet Quality Index for Children and Adolescents (KIDMED index), with many of them transitioning to a healthy weight status [23].

Both in Portugal and worldwide, various school-based interventions have successfully promoted healthier dietary habits and fostered a culture of health within school environments. Governments can play a pivotal role in supporting these initiatives, establishing food standards, and providing health education to encourage a modification towards healthier food choices. International guidelines highlight the responsibility of municipalities to improve children’s nutritional status, and they are essential participants in combating childhood obesity by creating supportive food and activity environments.

Several international programmes have demonstrated the effectiveness of school-based and multi-component interventions promoting healthy and sustainable eating habits. Beyond Europe, Canada’s Aliments du Québec and Équiterre fosters connections with local agriculture [24], while the United States’ National School Lunch Program increasingly aligns with sustainable sourcing [25]. In Japan, legislation requiring schools to employ nutritionists and prepare meals from scratch has contributed to some of the world’s lowest childhood obesity rates [26]. These experiences show that embedding healthy and sustainable eating into the school environment—through quality food provision, experiential learning, and community engagement—produces measurable improvements in children’s diets.

In Europe, similar initiatives have also been implemented. In France, the Programme National Nutrition Santé (PNNS) and EPODE (Ensemble Prévenons l’Obésité Des Enfants) involve local authorities, schools, and families in the promotion of healthy lifestyles [27,28]. Other examples include the United Kingdom’s Food for Life and Food Dudes programmes, which aim to improve children’s food choices and increase fruit and vegetable consumption [29,30], as well as Sweden’s policies on balanced school meals [31], Finland’s publicly funded school meal system [32], Denmark’s organic school meal strategy [33], Norway’s Fiskesprell programme, which promotes fish consumption among children [34], and Junior-Edu-Żywienie (JEZ), a Polish programme targeting nutrition education of children and parents [35]. In Portugal, the Sintra Grows Healthy programme illustrates how municipalities can integrate nutrition, physical activity and restrictions on exposure to unhealthy food marketing within school-based health promotion strategies [36]. These programmes reaffirm the need for school-based programmes that promote the MD while also addressing environmental sustainability. By integrating education on nutrition through practical food experiences and family and community engagement, schools can serve as a foundation for reversing unhealthy dietary trends and supporting the well-being of future generations. Schools are uniquely positioned to offer students a structured environment to learn about and adopt healthier behaviours that can have lifelong positive effects. Applying the principles of the MD within such frameworks can improve public health while fostering an understanding of crucial concepts such as food origins, seasonality, and environmental impact, supporting a healthier and more sustainable future [37].

Despite the growing recognition of the relationship between healthy dietary patterns and environmental sustainability, sustainability remains insufficiently incorporated into food-based dietary guidelines. It has been reported that only approximately 20% of European dietary guidelines explicitly integrate sustainability considerations [38]. This gap reinforces the need for educational strategies that connect nutritional recommendations with the environmental, cultural, and social dimensions of food choices. Food education can play a central role in this process by providing children and their communities with the knowledge and practical skills required to make informed, nutritionally appropriate, and more sustainable food choices [19,39]. Schools are particularly suitable settings for integrating these dimensions through experiential activities concerning dietary choices, seasonality, local food production, food storage, and food waste prevention.

In Portugal, school-based interventions integrating Mediterranean diet principles, sustainability education, food waste prevention, and experiential learning remain limited, particularly in primary school settings. In this context, the present intervention was developed under the Sustainable Food.LT Project, which is integrated into the National Network for Balanced and Sustainable Food (RNAES) and addresses the need for community-based strategies promoting healthier and more sustainable dietary habits from childhood [40]. The intervention aimed to promote Mediterranean diet principles and food literacy while introducing children to sustainability-related topics, including local and seasonal foods, food waste reduction, composting, food storage, and sustainable food production. The present study specifically aimed to describe the intervention and evaluate changes in adherence to the Mediterranean diet, assessed using the KIDMED index, among Portuguese primary school children.

## 2. Materials and Methods

The present community-based intervention was promoted by APRODER—Association for the Promotion of Rural Development of Ribatejo—in partnership with the Intermunicipal Community of Tagus Lezíria (CIMLT) and the Regional Coordination and Development Commission of Lisbon and Tagus Valley (CCDR-LVT). The project is part of the National Plan for Balanced and Sustainable Food (PNAES), includes 22 operations across Portugal, and is embedded in the National Network for Balanced and Sustainable Food (RNAES), funded by the Portuguese Recovery and Resilience Plan (PRR) and the European Union’s NextGenerationEU programme [40]. The Tagus Lezíria region was selected for this study because the project was territorially embedded in this intermunicipal community and implemented through the local institutional and school network.

### 2.1. Sampling and Participants

The target population comprised primary school students, aged between 6 and 13 years, enrolled in 23 schools across Tagus Lezíria, Portugal.

A total of 872 students participated in the educational intervention activities and are described in Table 1.

To evaluate the designed intervention programme, the Mediterranean Diet Quality Index for children and adolescents (KIDMED index) was used before and after the intervention [41,42]. This tool measures the adherence to MD guidelines through a questionnaire, allowing for assessment of the quality of children’s diets with a focus on MD principles, and is particularly useful for school-based programmes, as detailed in Section 2.4.

All 872 students’ parents/guardians were contacted and invited to provide a signed informed consent form authorizing their child’s interview. A total of 435 children from 19 schools had parental/guardian consent and were interviewed before the first intervention, completing the baseline KIDMED assessment. Of these 435 children, 370 remained available to be interviewed after the intervention, completing the post-intervention KIDMED assessment; these 370 children constituted the paired analytic sample used for the main outcome analysis.

### 2.2. Study Design

The intervention was developed in two rounds: the first one in the winter of 2024 and the second during the spring of the same year. The activities were carefully designed to align with the children’s age group and developmental stage, aiming to maximize engagement and retention. The key aspects considered in the development of activities included:

- Cognitive Appropriateness: Activities were tailored to match the cognitive abilities of the children, ensuring they could understand and engage with the concepts of healthy eating and sustainability.
- Engagement through Activity: By actively involving the children with dynamic and participatory activities, the programme aimed to foster enthusiasm and make learning about health and nutrition enjoyable.
- Locality and Seasonality of Materials: The materials, particularly the foods used in activities, were selected based on local availability and seasonality. This practice not only reinforced the knowledge of the principles of the MD, which emphasizes local and seasonal foods, but also introduced the children, in a practical way, to how to make sustainable food choices.
- Reproducibility at Home: Practical concepts that children could replicate at home were incorporated into the activities, supported by an activity book and resources designed to extend learning beyond the classroom. This approach helped to bridge learning from the school setting to everyday life, strengthening the knowledge and skills gained.

### 2.3. Activity Protocol

The intervention programme was designed to address school community needs, ensuring cultural relevance and reproducibility. It was implemented in two rounds (Table 1) with distinct focuses but shared goals: to promote healthy and sustainable eating, reduce food waste, and align with the MD principles in Portugal’s dietary guidelines [43]. All school-based activities were delivered by a multidisciplinary project team who shared responsibilities for implementing the educational activities and collecting field data.

**Table 1:** Overview of the two intervention rounds of the school-based intervention and their expected learning outcomes. Five activities were implemented in an autumn–winter session and later, in a spring–summer session.

	ACTIVITY	DESCRIPTION (DURATION)	SETUP & PROCEDURE	EXPECTED LEARNING OUTCOME
**Autumn–winter session**	**1: Telling a Story—** **Introduction to Healthy Eating** **and the MD**	Storytelling session introducing MD through a fictional narrative followed by an interactive segment with food cards (30 min).	Students explored the following food aspects: Healthiness, Regional Origin, and Personal Reflection on eating habits.	Enhanced understanding of local, healthy foods; MD principles; and the impact of dietary choices on health and the environment.
	**2: Sensory** **Exploration—** **Mystery Boxes** **with** **Seasonal Products**	Sensory activity with mystery boxes containing local MD foods (30 min).	Students identified foods by touch and smell, discussing Seasonality, Local Production, and Sustainability (embracing “ugly produce”).	Developed sensory awareness and appreciation for local, seasonal, and sustainable foods.
	**3: Healthy Plate—** **Building a** **Balanced and** **Sustainable Meal**	Students created balanced MD meals using a paper plate model (30 min) *.	Students used images of foods to create plates following MD proportions: 50% fruit/veg, 25% carbs, 25% protein (both animal- and plant-based).	Acquisition of practical knowledge of balanced meals and food group categorisation, reinforcing MD-aligned choices.
	**4: Composting—** **Understanding Waste Reduction and Soil Health**	Hands-on composting activity to learn about waste reduction (30 min).	Students categorized compost materials as “green” or “brown” and, where possible, applied compost to school gardens.	Fostered environmental awareness and understanding of waste reduction and soil health benefits.
	**5: Final Activity** **&** Materials *	Reflection session with take-home educational materials (30 min).	Students shared insights, received backpacks with materials (puzzle, recipes, family activity book).	Extended learning to home, encouraging sustainable food practices within families.
**Spring–summer session**	**1: Introduction to** **Sustainable Eating and Minimizing Food Waste**	Interactive discussion on sustainable eating and reducing food waste (30 min).	Students discussed practical strategies to reduce food waste at home and school.	Increased awareness of mindful consumption, food waste reduction, and sustainability.
	**2: Organize your Fridge, Minimize your Waste!**	Students learned efficient food storage practices (30 min).	Interactive dialogue between instructor and students to demonstrate fridge organization and storage tips to extend food shelf life and reduce waste.	Gained practical storage skills, promoting sustainability through efficient food management.
	**3: Sensory Exploration—Mystery Boxes with Seasonal Products**	Seasonal adaptation of Activity 2 of autumn–winter session with spring–summer produce (30 min).	Students explored sensory characteristics of seasonal foods.	Continued sensory engagement with local, seasonal produce, reinforcing awareness of seasonality.
	**4: Sowing—** **Introduction to** **Sustainable Food Production**	Planting activity focused on sustainable agriculture (30 min).	Students planted seeds, learning about soil preparation, sunlight, and sustainable agriculture practices.	Developed understanding of food production and sustainable practices, fostering a connection to nature.
	**5: Final Activity**	Reflection and commitment to sustainable practices (30 min).	Students shared personal action plans for conscious eating and sustainability.	Reinforced programme messages, encouraging long-term environmental responsibility.

* Education materials used during the session with children, take-home materials and materials produced for teachers are available online [44].

The activities described in Table 1 combine nutrition education, sensorial exploration, meal composition, composting, food storage, seed sowing and reflective tasks. The first round of the activities (the autumn–winter session) was conducted to reinforce healthy food habits and choices with a focus on MD. In the second round of activities (the spring–summer session), students explored sustainable eating practices with a specific emphasis on reducing food waste.

Along with this set of activities, educational materials were created for both children (including respective families) and teachers, enabling the replication of actions in the classroom and at home. These comprised activity guides for primary school students and teachers, educational materials on nutritional literacy and food waste prevention, and digital platforms to support the community.

All activity and educational resources were thoughtfully designed to encourage mindful eating habits, foster environmental awareness, and develop practical sustainability skills.

### 2.4. Evaluation of Intervention Effects Using KIDMED Index

The present school-based intervention was monitored using the KIDMED index to assess the programme’s effectiveness, evaluate improvements in dietary quality, and track progress toward sustainable eating habits. The KIDMED questionnaire consists of 16 yes-or-no questions, where children respond about dietary habits and points are added for healthy choices (e.g., eating fruit daily) and subtracted for unhealthy ones (e.g., skipping breakfast or consuming fast food). Scores can be categorized in three levels: (1) high adherence: ≥8; (2) medium adherence: 4–7; and low adherence: ≤3 [41]. The Portuguese validated version of the tool was used in this study [42]. Baseline KIDMED results collected before the intervention have been previously published [45] and were used as reference to evaluate the follow-up outcomes and the effectiveness of the current intervention action.

The implementation of the intervention and interviews with the children was approved by the Ethics Committee of IPSantarém under protocol nº 37/2023 (20 December 2023).

### 2.5. Statistical Analysis

All statistical analyses were performed using SPSS version 26. The nature of the variables under study and the lack of data normality and homoscedasticity, assessed by Kolmogorov–Smirnov and Levene’s tests, respectively, rendered the use of non-parametric tests as the best choice. All binary variables are presented as frequencies (%), while scale variable, age and KIDMED index are represented by mean ± standard deviation (mean ± sd). To compare participants’ responses to each question of the KIDMED questionnaire (yes/no) before and after the intervention, McNemar tests were employed; these non-parametric tests are used to analyze paired nominal data. All further analyses were performed considering each child individually, before and after the intervention (before autumn–winter session and after spring–summer session). The impact of the intervention, expressed as the percentage of children exhibiting an increase, no variation or decrease in the KIDMED index, was assessed. Subsequently, the degree of increase or decrease in the index was evaluated and analyzed considering the demographic characteristics. For all comparisons between groups exhibiting a decrease or an increase, Kruskal–Wallis tests were applied [46]. Differences were considered significant when *p* < 0.05.

## 3. Results

### 3.1. Demographic Characteristics of the Participants with Paired KIDMED Assessments

Of the 872 children participating in the present school-based intervention, a set of 370 (42% of the participants) were interviewed both before the first session and after the second session. The sociodemographic characterization of the respondents is shown in Table 2.

The intervention was monitored using the KIDMED index in a sample of children with a balanced proportion of boys and girls. Also, the proportion of children living in rural or urban areas was similar. The average age of the participants was 8.4 ± 1.1, with children aged 7, 8, 9 or 10 years old predominating, exhibiting percentages of 14.4, 26.4, 37.3 and 14.7, respectively (Table 2).

### 3.2. Variation in the Adherence to the Mediterranean Diet

**Figure 1:**
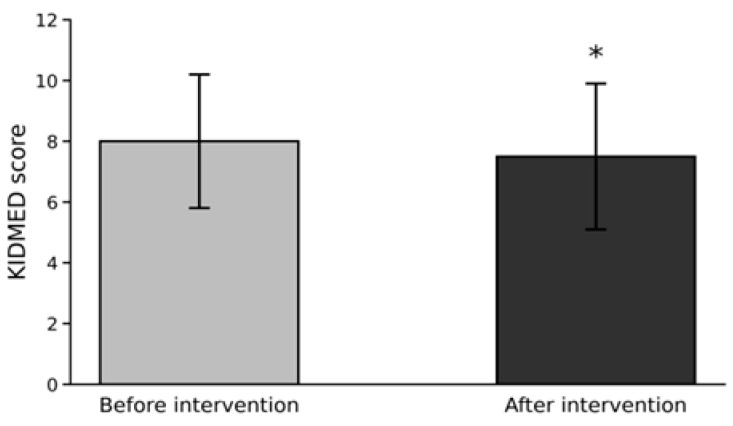
Participants’ KIDMED score before and after the intervention (N = 370). Data are expressed as mean ± standard deviation. Differences between measurement points were assessed using the Wilcoxon matched-pairs signed-rank test (two samples). Statistical significance was set at *p* < 0.05, with * indicating a significant difference compared to baseline (before the intervention).

At the end of the interventions, the mean KIDMED score was compared with the one before the interventions, revealing a decrease from 8.0 ± 2.2 to 7.5 ± 2.4 (Figure 1).

Table 3 summarizes the paired before-and-after responses to each KIDMED item. Positive changes were observed in breakfast consumption, lower fast-food intake, lower sweet and pastry consumption, and slightly higher fish and olive oil use. In contrast, fruit, vegetable, pulse, and nut consumption did not improve.

**Figure 2:**
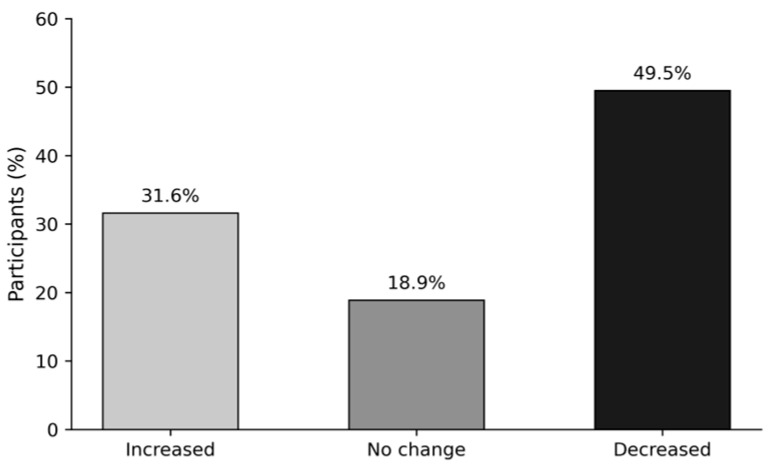
Individual change in KIDMED score after the intervention. Percentages indicate participants who improved their score, showed no change, or decreased their score (N = 370).

At the individual level, 31.6% of the participants improved their KIDMED score after the intervention, 18.9% showed no change and 49.5% decreased their level of adherence (Figure 2).

Considering individual changes according to sociodemographic characteristics, KIDMED scores improved by 29.9% of boys and 33.0% of girls, whereas decreases of 54.0% and 45.3% were observed, respectively. Children aged 10 years showed the highest proportion of improvement (41.8%). The proportions of children showing improvement were almost identical among rural and urban participants (31.5% and 31.3%, respectively) (Table 4).

Looking at the analyzed boys and girls whose KIDMED score increased after the intervention, Table 4 reveals the proportions to be 29.9% of boys and 33.0% of girls. Among the overall sample of students, more than 20% of them increased adherence to MD, which is particularly relevant considering the Portuguese national target of increasing adherence to the MD [47]. Although the overall KIDMED score decreased significantly after the intervention (Figure 1), it remained relatively high and several item-level behaviours improved (Table 3).

## 4. Discussion

This study evaluated the effects of a school-based intervention integrating MD education with sustainability-related activities among Portuguese primary school children. Although the mean KIDMED score decreased significantly from 8.0 ± 2.2 at baseline to 7.5 ± 2.4 after the intervention, individual responses were heterogeneous: 31.6% of the children improved their KIDMED score, 18.9% showed no change, and 49.5% experienced a decrease. This apparent discrepancy reflects the multidimensional nature of the KIDMED index. Although several dietary behaviours improved, these positive changes were insufficient to compensate for less favourable changes in other positively scored KIDMED components, resulting in an overall decline in mean MD adherence at the group level.

The relatively high baseline adherence to the MD is an important consideration when interpreting these findings. In a previously published analysis of the complete baseline sample, 64.6% of the children were classified as having high adherence to MD [45]. Even higher levels of adherence have been reported in other Portuguese primary school populations. For example, Marques et al. found that 77.6% of children aged 9–11 years attending schools in Porto and Maia exhibited high MD adherence [48]. Such differences across studies may reflect variations in age, geographical location, school environment, family characteristics, socioeconomic status, and the timing of data collection. The high baseline dietary profile observed in the present study may have reduced the potential for further improvement among some participants. Nevertheless, although a ceiling effect may have limited the potential for further improvement, it is unlikely to fully explain the significant decline observed in the overall KIDMED score.

The item-level analysis helps explain this apparent contradiction. Significant improvements were observed in individual KIDMED items related to fish consumption, olive-oil use, breakfast consumption, reduced frequent attendance at fast-food restaurants, lower consumption of commercial pastries at breakfast, and less frequent consumption of sweets.

The most favourable result concerned breakfast. For question 12, the proportion of children who did not skip breakfast increased from 79.8% to 88.6%, corresponding to an increase of 8.8%. This result is relevant because regular breakfast consumption during childhood has been associated with better overall diet quality, more favourable nutrient intake, healthier weight-related outcomes, improved cognitive performance, and better school achievement [49]. Nevertheless, the improvement concerned breakfast frequency rather than its nutritional quality. The proportions consuming cereals or cereal products and dairy products at breakfast, assessed through questions 9 and 13, decreased. Future interventions should therefore address not only whether children eat breakfast but also its nutritional composition.

Favourable changes were also observed in the frequency of fast-food restaurant attendance (question 6). The proportion of children reporting that they did not attend fast-food restaurants more than once a week increased from 85.4% at baseline to 89.6% post-intervention. Smaller improvements were also observed in lower consumption of commercial pastries at breakfast and sweets consumed several times per day (questions 14 and 16). These findings suggest that dietary behaviours explicitly and repeatedly reinforced through school-based educational activities may be more responsive to short-term intervention. Nevertheless, given the absence of a control group, these behavioural changes should be interpreted cautiously and cannot be attributed exclusively to the intervention.

In contrast, several core components of the MD declined. Daily fruit or fruit juice consumption decreased 4.7%, consumption of a second daily serving of fruit decreased 10.8%, daily vegetable consumption decreased 5.0%, and vegetable consumption more than once a day decreased 11.5%. Pulse consumption decreased 16.5%, and regular nut consumption decreased 5.0%. Together with smaller declines in cereal- and dairy-related items (1.2% and 12.6%, respectively), these changes contributed to the reduction in the overall KIDMED score.

Low fruit and vegetable consumption among children and adolescents remains an international public health concern. Data from the 2021/2022 WHO Health Behaviour in School-aged Children survey showed that fewer than 40% of adolescents consumed fruit or vegetables daily and that consumption declined with age [50]. These foods depend strongly on household availability, parental purchasing decisions, meal preparation, repeated exposure, and children’s acceptance. School-based educational interventions may consequently have limited capacity to modify their consumption without sustained support from families and the home food environment.

The sociodemographic findings should be interpreted descriptively and cautiously. Girls showed a more favourable pattern than boys, although the data do not establish a sex-specific intervention effect. Age appeared to be more relevant: 41.8% of the children aged 10 years improved their KIDMED score, whereas 57.9% of the six-year-old children showed a decrease. Older primary school children may have greater cognitive maturity and a better capacity to understand and independently apply nutrition-related messages. Conversely, younger children remain highly dependent on parents and caregivers for food purchasing, availability, preparation, and family meal routines. They may also have greater difficulty interpreting questionnaire items concerning consumption frequency and number of servings. As parental practices and children’s comprehension of the questionnaire were not directly evaluated, these explanations should be regarded as plausible hypotheses rather than demonstrated mechanisms.

The findings according to place of residence do not support the conclusion that urban children improved more than rural children. In the previously published baseline analysis, high MD adherence was observed in 60.5% of rural children and 70.2% of urban children, although the difference was not statistically significant [45]. In the paired sample, the proportions showing improvement were almost identical among rural and urban children, at 31.5% and 31.3%, respectively. A decrease was observed in 47.4% of rural children and 52.4% of urban children. The inconsistent rural–urban patterns reported in the literature further indicate that place of residence should not be interpreted independently of family, socioeconomic, cultural, and food-environment factors [16].

Socioeconomic characteristics may influence both baseline adherence and responses to educational interventions. Household income, parental education and employment, food affordability, and access to diverse foods can shape children’s dietary choices and the home food environment. However, these variables were not collected in the present study, and their influence could not be assessed. The absence of socioeconomic information is therefore an important limitation that should be addressed in future research.

The intervention integrated MD education with practical activities concerning local and seasonal foods, sensory exploration, food storage, food waste prevention, composting, and seed sowing. The MD is recognized as a healthy and sustainable dietary pattern with nutritional, environmental, cultural, and social dimensions [2,4,38,51]. Accordingly, KIDMED provides information about adherence to several dietary components that are compatible with a sustainable diet. However, it does not directly assess food waste, composting, selection of local or seasonal foods, sustainable food production, or environmental awareness. The present study can therefore evaluate changes in MD adherence but cannot determine whether these additional sustainability-related outcomes improved.

Although knowledge and perceptions were not directly assessed, evidence from educational interventions suggests that changes in knowledge, awareness, and attitudes may precede measurable and sustained changes in dietary behaviour [19,52]. The sensory exploration of seasonal foods, composting, food storage, and seed-sowing activities provided practical experiences connecting food choices with food production, seasonality, and waste prevention. These activities are consistent with the broader environmental and cultural dimensions of the MD [2,4,51]. Their effects on children’s knowledge, perceptions, and practices should nevertheless be assessed using specific instruments in future studies.

The limited direct involvement of families may have reduced the intervention’s capacity to influence dietary habits outside school. Children’s adherence to the MD is strongly shaped by parental dietary practices, food availability at home, family mealtime routines, and the broader household food environment [53,54,55]. Although take-home educational materials and family activities were provided, parental participation was not monitored or systematically incorporated into the intervention. This limitation may have been especially important for younger children. Portuguese initiatives such as Eat Mediterranean [56] and Sintra Grows Healthy [36] illustrate the value of combining school actions with family, health-professional, community, and municipal involvement. More broadly, systems-based approaches connecting families, schools, communities, and local institutions may provide a more supportive environment for the adoption and maintenance of healthier dietary practices [57].

Intervention duration may also have influenced the results. Dietary habits develop within family, school, social, and cultural contexts and may be resistant to change after a limited number of sessions. Longer school-based programmes suggest that repeated and sustained interventions may be required to consolidate nutrition-related knowledge and translate it into stable dietary behaviours [58]. Family-centred approaches also indicate that continued parental engagement is important for maintaining behavioural changes during childhood [54]. Short-term programmes may introduce concepts and encourage selected favourable behaviours, but they may provide insufficient reinforcement to improve overall MD adherence.

Overall, the intervention was followed by mixed dietary changes. The improvement in breakfast consumption and the reduction in selected less healthy behaviours are relevant, but they occurred alongside a significant decrease in the overall KIDMED score and reductions in several core components of the MD. Future interventions should combine school-based activities with stronger parental participation, repeated reinforcement, and strategies adapted to children’s ages and family contexts. Longer controlled studies should incorporate socioeconomic information and direct measures of food literacy and sustainability-related knowledge and practices. Tailored strategies, greater family involvement, and extended duration are key elements to maximize the effectiveness and scalability of future interventions.

## 5. Conclusions

This study evaluated a two-season community-based school intervention integrating Mediterranean diet education with participatory activities related to seasonal and local foods, sensory exploration, balanced meals, food storage, composting, food waste prevention, and sustainable food production.

Overall adherence to the MD did not improve following the intervention. The mean KIDMED score decreased significantly from 8.0 ± 2.2 to 7.5 ± 2.4, and 49.5% of participants showed a reduction in their individual score. Nevertheless, the response was heterogeneous: 31.6% of the children improved their KIDMED score, and favourable changes were observed in selected behaviours, particularly regular breakfast consumption and avoidance of frequent fast-food consumption. Smaller favourable changes were also observed in the consumption of fish and olive oil and in the avoidance of pastries and sweets. Conversely, fruit, vegetable, pulse, and nut consumption did not improve and contributed to the mixed overall outcome.

The findings demonstrate the difficulty of changing dietary habits through a relatively short school-based intervention, particularly when children’s food choices remain strongly influenced by their families and home food environments. The results also indicate that improvements in selected behaviours do not necessarily translate into an improvement in the overall KIDMED score.

Future interventions should provide repeated reinforcement, address both the frequency and nutritional quality of meals such as breakfast, and place greater emphasis on core MD foods that did not improve. They should also incorporate structured parental participation and evaluate socioeconomic, food literacy, and sustainability-related outcomes using appropriate instruments. Tailored strategies, greater family involvement, and extended duration are key elements to maximize the effectiveness and scalability of future interventions.

Schools remain strategic settings for connecting nutrition education, Mediterranean food culture, and sustainability. Achieving sustained dietary change is likely to require long-term and multi-component approaches involving schools, families, communities, health professionals, and local authorities.

## Figures and Tables

**Table 2:** Sociodemographic characteristics of the interviewed children with paired KIDMED assessments (*n* = 370). Data are expressed as number of participants and percentages, *n* (%), except for age, which is presented as mean ± standard deviation (mean ± SD). Percentages are rounded to one decimal place.

**Gender**, *n* (%)	***n***	**%**
Boys	171	46.2
Girls	199	53.8
**Age** (mean ± SD)	8.4 ± 1.1	
**Age categories**, *n* (%)	***n***	**%**
6 years	19	5.1
7 years	53	14.4
8 years	98	26.4
9 years	138	37.3
10 years	54	14.7
11 years	6	1.6
12 years	1	0.3
13 years	1	0.3
**Residence**, *n* (%)	***n***	**%**
Rural	216	58.5
Urban/town/village	154	41.5

**Table 3:** Participants’ answers before and after the school’s interventions, according to their alignment with MD principles, to each question of the KIDMED questionnaire. Data are expressed as percentages (%).

KIDMED Question		Answer	Before Intervention (%)	After Intervention (%)	*p*-Value
1	Fruit or fruit juice daily	Yes	87.6	82.9	<0.001
2	Second serving of fruit daily	Yes	61.6	50.8	0.013
3	Fresh or cooked vegetables daily	Yes	89.5	84.5	<0.001
4	Fresh or cooked vegetables more than once a day	Yes	58.2	46.7	0.376
5	Regular fish consumption (at least 2–3 times/week)	Yes	67.1	67.6	<0.001
6	Fast-food restaurant more than once a week	No	85.4	89.6	<0.001
7	Pulses more than once a week	Yes	56.8	40.3	0.457
8	Pasta or rice almost daily (≥5 days/week)	Yes	90.0	85.1	<0.001
9	Cereals or cereal products for breakfast	Yes	84.1	82.9	<0.001
10	Regular consumption of nuts (at least 2–3 times/week)	Yes	20.5	15.5	<0.001
11	Use of olive oil at home	Yes	92.4	93.2	<0.001
12	No breakfast	No	79.8	88.6	<0.001
13	Dairy product at breakfast	Yes	87.3	85.1	<0.001
14	Commercial baked goods or pastries for breakfast	No	85.7	86.7	<0.001
15	Two cups of milk/yoghurt and/or 40 g of cheese daily	Yes	69.8	59.4	<0.001
16	Sweets and candy several times a day	No	88.1	89.6	<0.001

KIDMED index (Mediterranean Diet Quality Index to evaluate MD adherence among children and adolescents). Criteria to meet MD principles are shown for each question as “yes” or “no”.

**Table 4:** Changes in children’s KIDMED index after the intervention, according to their sociodemographic characteristics. Data are relative in % to the total sample of each sociodemographic characteristic.

	Decrease	No Variation	Increase
**Gender** (%)			
Boys	54.0	16.1	29.9
Girls	45.3	21.7	33.0
**Age categories** (%)			
6 years	57.9	10.5	31.6
7 years	48.1	16.7	35.2
8 years	53.5	19.2	27.3
9 years	47.9	22.9	29.3
10 years	41.8	16.4	41.8
**Residence** (%)			
Rural	47.4	21.1	31.5
Urban/town/village	52.4	16.3	31.3

## Data Availability

The data supporting the findings of this study are available from the corresponding authors upon reasonable request.

## Abbreviations

The following abbreviations are used in this manuscript:

MD	Mediterranean Diet
MDP	Mediterranean Dietary Pattern
KIDMED index	Mediterranean Diet Quality Index for Children and Adolescents
NCDs	Non-Communicable Diseases
ADHD	Attention Deficit Hyperactive Disorder
